# Upregulation of miR‐200a and miR‐204 in MPP^+^‐treated differentiated PC12 cells as a model of Parkinson’s disease

**DOI:** 10.1002/mgg3.548

**Published:** 2019-02-03

**Authors:** Maryam Talepoor Ardakani, Mahsa Rostamian Delavar, Masoud Baghi, Mohammad Hossein Nasr‐Esfahani, Abbas Kiani‐Esfahani, Kamran Ghaedi

**Affiliations:** ^1^ Department of Biology, School of Sciences University of Isfahan Isfahan Iran; ^2^ Department of Cellular Biotechnology, Cell Science Research Center Royan Institute for Biotechnology, ACECR Isfahan Iran

**Keywords:** differentiated PC12 cell, miR‐200a, miR‐204, MPP^+^, Parkinson’s disease

## Abstract

**Background:**

Parkinson's disease (PD) is ranked as the second most common neurodegenerative disorder caused by loss of dopaminergic neurons in the substantia nigra. Micro(mi)RNAs are a class of small noncoding RNAs that regulate gene expression and aberrant expression of them is closely correlated with many neurodegenerative conditions including PD. Silent information regulator 1 (*SIRT1*) as a known deacetylase and B‐cell lymphoma‐2 (*BCL2*) as an antiapoptotic factor play vital roles in neural protection and survival.

**Methods:**

Differentiated PC12 cells exposed to MPP^+^ were served here as a known PD model. Cell viability was determined by MTS assay. Apoptotic cells and ROS levels were detected using flow cytometry. Gene selection and miRNA–mRNA interaction analysis were performed through in silico methods. Relative expression of miRNAs and genes was examined by RT‐qPCR.

**Results:**

MPP^+^ exposure markedly reduced cell viability, enhanced oxidative stress, and induced apoptosis in differentiated PC12 cells. *Sirt1* and *BCL2*were shown to be markedly declined in response to MPP^+^, while miR‐200a and miR‐204 were significantly upregulated.

**Conclusion:**

The first novel finding of the current study is altered expression of miR‐200a and miR‐204 in differentiated PC12 cells in response to MPP^+^, suggesting that deregulation of them participate in MPP^+^ neurotoxicity mechanisms, possibly via affecting the expression of *Sirt1* and *BCL2* as potential targets.

## 1 INTRODUCTION

Parkinson's disease (PD) is ranked as the second most common neurodegenerative disease after Alzheimer's disease (AD) characterized by the death of dopaminergic neurons in the substantia nigra as the main pathological hallmark (Farshbaf et al., 2016). Although the precise etiology of PD is still unclear, protein misfolding, neuroinflammation, mitochondrial dysfunction, and oxidative stress have all been found as associated pathogenic processes with the loss of dopaminergic neurons (Farshbaf et al., 2016; Kanagaraj, Beiping, Dheen, & Tay, 2014; Kim et al., 2007).

Micro(mi)RNAs are small noncoding RNAs (about 20–24 nucleotides) that repress gene expression by binding to the 3′UTR of target mRNAs and block translation or promote degradation of them (Delavar et al., 2018; Kanagaraj et al., 2014). miRNAs have been recently suggested as biomarkers or therapeutic targets for neurodegenerative diseases due to their vast regulatory potential (Johnson, Noble, Tartaglia, & Buckley, 2012). According to previous studies, miRNAs are relevant to neurodegenerative processes and aberrant expression of specific miRNAs has been reported in different PD models and human brain samples (Maciotta, Meregalli, & Torrente, 2013; Sonntag, 2010). Although there is convincing evidence supporting miRNAs involvement in PD occurrence and progression, few surveys have been conducted to identify deregulated miRNAs in this condition (Kanagaraj et al., 2014).

Moreover, there are PD‐linked neuroprotective genes which their expression can be influenced by perturbed miRNA machinery. Silent information regulator 1 (*SIRT1*) is a nicotinamide adenine dinucleotide (NAD^+^)‐dependent deacetylase which participates in neural protection against oxidative stress and apoptosis by modulating a wide variety of substrates, for example, p53, PGC‐1α, and FoxO (Kim et al., 2007; Pallas et al., 2008). *SIRT1* is a master modulator of mammalian transcription in response to biological stresses and implicated in an evolutionarily conserved protective pathway which enables organisms to deal with such adversities (Kim et al., 2007).

B‐cell lymphoma‐2 (*BCL2*) is a crucial antiapoptotic protein which inhibits cytochrome c translocation to the cytosol, prevents caspases activation and finally blocks apoptosis (Yang et al., 1998; Zheng, Liu, Fan, Shi, & Zhang, 2016). Overexpression of the BCL2 protein in multiple PD models provides protection against cell death induced by neurotoxins. *BCL2* promotes neuronal survival by mechanisms that may include both antiapoptotic functions and oxidative stress inhibition (Yang et al., 1998).

1‐Methyl‐4‐phenylpyridinium (MPP^+^) is a widely used neurotoxin which induces PD‐related alterations in mitochondrial activity such as complex I inhibition and enhances the reactive oxygen species (ROS) production (Delavar et al., 2018; Farshbaf et al., 2016). PC12 cells originated from rat pheochromocytoma could be differentiated into neuron‐like cells in response to nerve growth factor (NGF). Accordingly, MPP^+^‐treated differentiated PC12 cells as a cellular model for PD research were utilized here (Farshbaf et al., 2016; Lipman, Tabakman, & Lazarovici, 2006).

Collectively, regarding that perturbed miRNA/mRNA expression networks can be considered as a mechanism in neurodegeneration (Sonntag, 2010), the aim of the current study is to identify some altered genes and miRNAs in the culture model of PD. We selected two PD‐related neuroprotective genes and two targeting miRNAs, miR‐204, and ‐200a which were never studied or focused on in cellular PD models before for the present study.

## 2 MATERIALS AND METHODS

### 2.1 In silico methods

Through the literature survey, deregulated genes and miRNAs in different neurodegenerative conditions were identified. TargetScan 7.1 (Agarwal, Bell, Nam, & Bartel, 2015) and miRWalk 2.0 (Dweep & Gretz, 2015), two more inclusive databases for Rat organism, were employed to predict targeting miRNAs of selected genes. Additionally, DianaTools MirPath v.3 was recruited to visualize the signaling pathways in which miR‐200a and miR‐204 are implicated. Pathways related to genes were gathered from KEGG (Kanehisa, Sato, Kawashima, Furumichi, & Tanabe, 2016), BIOCARTA (http://www.biocarta.com) and PANTHER (Mi et al., 2017). Signaling pathway enrichment analysis was conducted by imputing selected genes symbols in the DAVID online database, version 6.8 (Huang, Sherman, & Lempicki, 2008). Through DisGeNET v3.0 database (http://www.disgenet.org/web/DisGeNET), a set of 100 genes strongly associated with PD was obtained. In next step, the interactions of selected genes were assessed by STRING‐db (Szklarczyk et al., 2014) and visualized by Cytoscape 3.6.0 software. Moreover, to evaluate the expression of these genes in different regions of brain, we used Genevestigator which is an available microarray database (https://www.genevestigator.com).

### 2.2 Cell culture and differentiation

PC12 cell line was obtained from Pasteur Institute of Iran (Tehran, Iran), and cultured on poly‐l‐ornithine (Sigma, USA) and laminin (Sigma)‐coated dishes in high‐glucose Dulbecco's modified Eagle's medium (DMEM; Gibco, USA) supplemented with 10% (v/v) heat‐inactivated horse serum (Sigma), 5% (v/v) heat‐inactivated fetal bovine serum (Gibco), and 100 U/ml penicillin–streptomycin (Gibco) at 37°C under a humidified atmosphere of 5% CO_2_. To induce differentiation, cells were treated for 7 days in medium containing 50 ng/ml of NGF‐β (Cell Guidance Systems, USA), 100 U/ml penicillin/streptomycin and 1% (v/v) horse serum. The half volume of differentiating medium was refreshed every 2 days.

### 2.3 Cell survival evaluation

Cell viability was determined by MTS assay. The mitochondrial dehydrogenase activity reduces 3‐(4, 5‐dimethylthiazol‐2‐yl)‐5(3‐carboxy methoxyphenyl)‐2‐(4‐sulfophenyl)‐2H‐tetrazolium (MTS) to the soluble formazan product in the presence of phenazine methosulfate (PMS). For cytotoxicity assay, PC12 cells were seeded at the density of 1 × 10^4^ cells/well in 96‐well plate dishes and differentiated. Twenty‐four hours before neurotoxin treatment, the medium was changed to low‐serum medium. Then, cells were treated with various concentrations of MPP^+^. After 24 hr, 20 μl of MTS/PMS solution (Promega, USA) was added to each well and incubated for 3 hr at 37°C. The absorbance of formazan product at 490 nM was measured by a spectrophotometer (Awareness model, USA).

### 2.4 Measurement of intracellular ROS production

Intracellular ROS was measured by dichlorodihydrofluorescein diacetate (DCFH‐DA) oxidation. DCFH‐DA passes into the cytosol and is deacetylated by nonspecific esterases to nonfluorescent DCFH. The intracellular ROS oxidizes DCFH into fluorescent dye 2,7‐dichlorofluorescin (DCF). To measure ROS, 4 × 10^5^ cells/well in 6‐well plate dishes were differentiated and treated with MPP^+^ and then were incubated with 0.5 μM DCFH‐DA (Sigma) for 15 min. Fluorescence intensity was detected at an excitation wavelength of 485 nm and an emission wavelength of 530 nm using a FACSCalibur flow cytometer (Becton–Dickinson, USA).

### 2.5 Flow cytometry analysis of cell apoptosis

Phosphatidylserine (PS) is translocated from the internal to the external membrane surface when cells are undergoing apoptosis. This redistribution of PS is considered as an indicator of early apoptosis. Annexin V and its conjugates can be used for detection of apoptosis because they interact strongly and specifically with exposed PS. For measuring external PS, 4 × 10^5^ PC12 cells were differentiated on 6‐well plates and treated with MPP^+^. Then, the cells were incubated with 10 μl of FITC‐Annexin V (IQ Products, the Netherlands) for 20‐min in the dark at 4°C. Finally, the samples were analyzed by FACSCalibur flow cytometer (Becton Dickinson).

### 2.6 RNA isolation and real‐time PCR

Total RNA was isolated with Trizol (Invitrogen, USA) according to the manufacturer's instructions. cDNA synthesis for miRNAs was performed by using the miR‐Amp kit (Parsgenome, Iran) in poly (A) tailing manner. The expression levels of miRNAs were assessed through SYBR green method and monitored by ABI PRISM 7500 instrument (Applied Biosystems, USA). As a reference gene, U6 snRNA was used for normalization of miRNAs expression. cDNA synthesis for *Sirt1* and *BCL2* was done by PrimeScript™ RT reagent Kit (Takara, Japan) using random hexamer primers. RT‐qPCR was done on ABI PRISM 7500 instrument (Applied Biosystems) using specific primer pairs. The forward (F) and reverse (R) primers for the specific amplification of *Sirt1* were, F: 5' AAGGAGCAGATTAGTAAGC 3' and R: 5' TAGAGGATAAGGCGTCAT 3'. The primer pairs for *BCL2* were F: 5' ACTTCTCTCGTCGCTACCGTC 3' and R: 5' AAGAGTTCCTCCACCACCGT 3'. The primer pairs for *Gapdh* were F: 5' TGCCGCCTGGAGAAACC 3' and R: 5' TGAAGTCGCAGGAGACAACC 3' (Macrogen Company, South Korea). The relative expression of target genes was normalized by Glyceraldehyde 3‐phosphate dehydrogenase (*Gapdh*) as a reference gene. All reactions were performed in triplicate. Real‐time data were analyzed based on 2^−ΔΔCT^ method.

### 2.7 Statistical analysis

All statistical analyses were performed using SPSS 22.0. Data are presented as mean ± SD from three independent experiments. Student's independent‐samples *t* test was used to examine significant differences between MPP^+^‐treated and control cells. Differences were considered statistically significant at *p* < 0.05.

## 3 RESULTS

### 3.1 Gene selection

**Figure 1:**
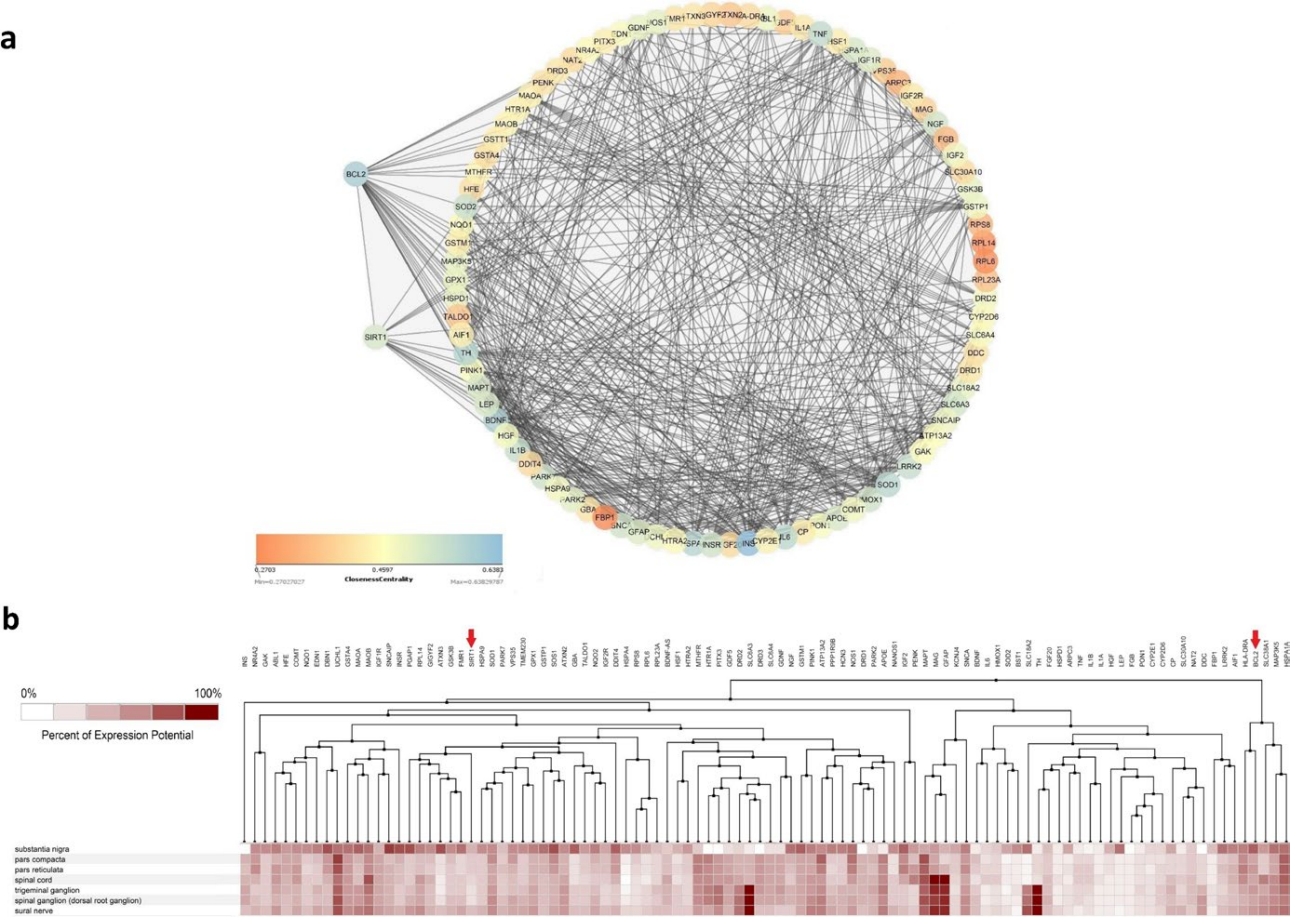
Interactions between PD‐related genes and their expression pattern in various brain regions. (a) One hundred PD‐related genes were retrieved from the Disgenet database and inputted to STRING‐db. Then, interactions between genes including *SIRT1* and *BCL2* were visualized in Cytoscape. Interestingly, in addition to their interaction with other PD‐related genes, *SIRT1* and *BCL2* are also connected to each other. (b) Expression analyses of the PD‐related genes including *SIRT1* and *BCL2* in different brain regions by Genevestigator

To select proper genes, first, deregulated genes in neurodegenerative disease and models were retrieved via literature survey. Then, inputting official gene symbols of chosen mRNAs into various databases, associated pathways with these deregulated genes in BIOCARTA, KEGG, and PANTHER were acknowledged. Among all deregulated genes, we selected *SIRT1* and *BCL2* which are enriched in pathways relevant to neurodegeneration and MPP^+^ mechanisms, for example, apoptosis or oxidative stress in accordance with Table 1 data. Additionally, combined schematic KEGG signaling pathways related to *SIRT1* and *BCL2* (retrieved from DAVID) are represented in Supporting information Figures S1 and S2. Besides, according to Figure 1a, there are strong interactions between *SIRT1*/*BCL2* and other PD‐associated genes. Interestingly, in addition to their interaction with other PD‐related genes, *SIRT1* and *BCL2* are also connected to each other (Figure 1a). Ultimately, performing a Genevestigator analysis, we gained insight into expression patterns of these PD‐related genes in various brain regions including the substantia nigra (Figure 1b).

**Table 1:** Signaling pathway related to *SIRT1* and *BCL2* genes collected from different databases. *SIRT1* and *BCL2* genes appear to be implicated in many critical signaling pathways associated with neurodegeneration, oxidative stress, and apoptosis

Gene	Database	Pathway name
*SIRT1*	KEGG	FOXO signaling pathway
		Longevity regulating pathway
	REACTOME	Cellular responses to stress
		Epigenetic regulation of gene expression
	PANTHER	p53 pathway
*BCL2*	KEGG	Amyotrophic lateral sclerosis (ALS)
		Neurotrophin signaling pathway
		Apoptosis
	REACTOME	Intrinsic pathway for apoptosis
	PANTHER	Huntington disease
		p53 pathway

### 3.2 miRNA selection

**Figure 2:**
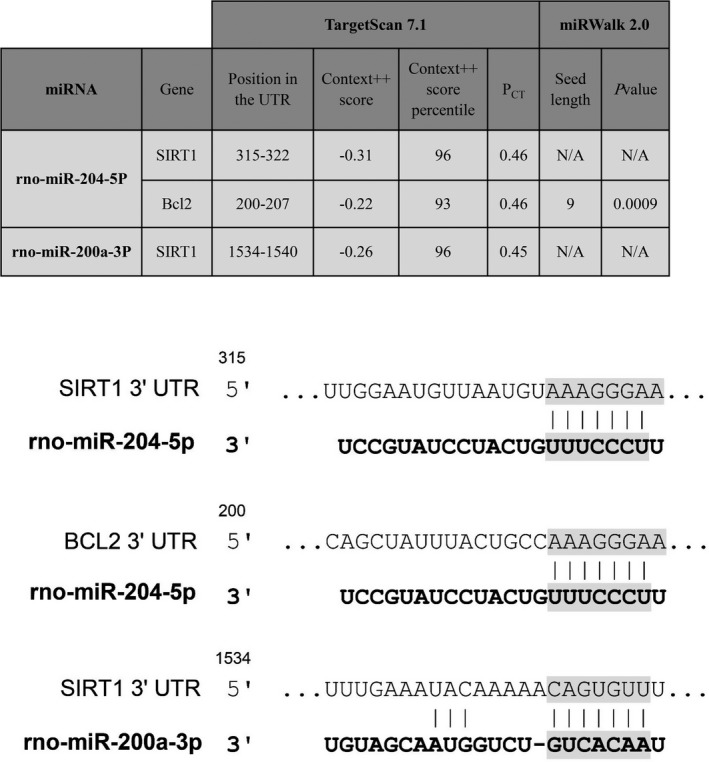
Predicted miRNA–mRNA interactions. (a) Predicted miRNA–mRNA interactions were taken from miRwalk 2.0 and TargetScan 7.1. Based on results predicted by these two algorithms indicating a strong possibility of miRNA–mRNA interactions, miR‐200a, and miR‐204 were selected as candidate miRNAs for the present study. (b) The potential matching positions of miR‐200a‐3p and miR‐204‐5p within the 3′‐UTR of *SIRT1* and *BCL2* predicted by Targetscan are shown in the illustration

**Figure 3:**
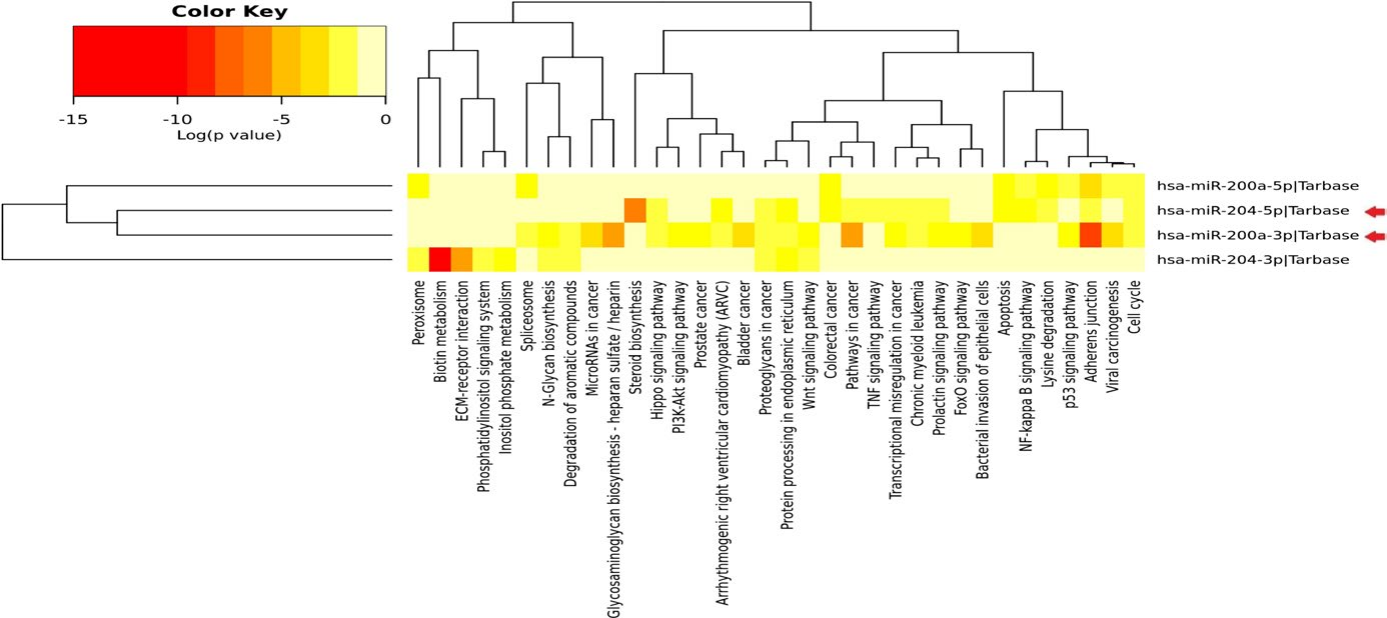
Heatmap view of miR‐200a‐ and miR‐204‐related signaling pathways. The heatmap demonstrates miR‐200a‐ and miR‐204‐associated pathways according to DIANA miRPath v.3. Here, p‐value marks the inspected signaling pathways which are significantly enriched with miRNAs targets. Additionally, color gradient represents pathway value with red, as the highest importance, and pale yellow, as the lowest

To choose appropriate miRNAs, first, literature mining was conducted to identify altered miRNAs in neurodegenerative conditions and models. Then, miRwalk 2.0 and TargetScan 7.1 were employed for miRNA‐target predictions to identify possible interactions between miRNAs and selected genes. Among these literature‐derive altered miRNAs, miR‐200a, and miR‐204 with the strong possibility to interact with selected genes have been picked out as ultimate candidate miRNAs for the present study (Figure 2). Interestingly, miR‐200a and miR‐204 are highly relevant to crucial PD‐related signaling pathways including P53 signaling pathway, apoptosis, and FoxO signaling pathway as shown in the heatmap retrived from DIANA miRPath v.3 (Figure 3).

### 3.3 MPP^+^‐induced cell viability loss

**Figure 4:**
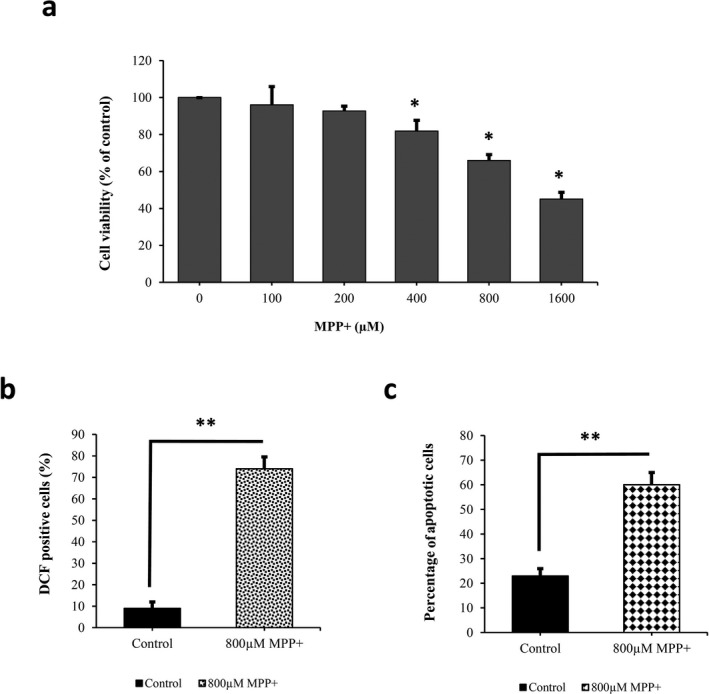
MPP^+^‐induced cell viability decline, ROS overproduction, and apoptosis in differentiated PC12 cells. (a) Differentiated PC12 cells were exposed to various concentrations of MPP^+^ for 24 hr, and cell viability was evaluated by MTS assay. 800 μM MPP^+^ which caused 34.2% cell death was selected as an appropriate concentration for following experiments. (b) Flow cytometric analysis of ROS production through DCFH‐DA staining showed that treating differentiated PC12 cells with MPP^+^ caused a sevenfold increase in percentages of DCF‐positive cells demonstrating an elevation in ROS generation. (c) Flow cytometric detection of apoptotic cells using Annexin V‐FITC staining represented that MPP^+^ exposure markedly enhanced apoptotic rate in differentiated PC12 cells. (**p < 0.01, *p < 0.05 vs. control, independent‐samples *t* test)

Here, MPP^+ ^was used to induce oxidative stress and neurotoxicity in differentiated PC12 cells. In order to obtain the appropriate concentration of MPP^+^, differentiated PC12 cells were treated with different concentrations of MPP^+^ (100, 200, 400, 800, 1,600 μmol/L) for 24. Results from MTS assay have shown a concentration‐dependent decrease in cell viability following MPP^+^ treatment. Finally, 800 μM MPP^+^ as an optimal concentration was selected for subsequent experiments which induced 34.2% cell death in differentiated PC12 cells (Figure 4a).

### 3.4 MPP^+^‐induced ROS overproduction

Dichlorodihydrofluorescein diacetate assay was used to measure intracellular ROS production in differentiated PC12 cells before and after MPP^+^ treatment. According to flow cytometry data, treating differentiated PC12 cells with MPP^+^ caused a significant increase in DCF‐positive cell numbers demonstrating an elevation in ROS production. As shown in Figure 4b, approximately sevenfold increase in the percentage of DCF‐positive cells occurred after MPP^+^ treatment compared to untreated cells.

### 3.5 MPP^+^‐induced apoptosis

Annexin V‐FITC staining was used to detect apoptotic cells by flow cytometry. According to Figure 4c, only 23% of cells were positive for Annexin V–FITC binding in the control group. Following MPP^+^ exposure, the percentage of positive cells was 60% representing MPP^+^ treatment considerably enhanced apoptotic rate in differentiated PC12 cells (Figure 4c).

### 3.6 Downregulated mRNA levels of *Sirt1 *and *BCL2* after MPP^+^ treatment

**Figure 5:**
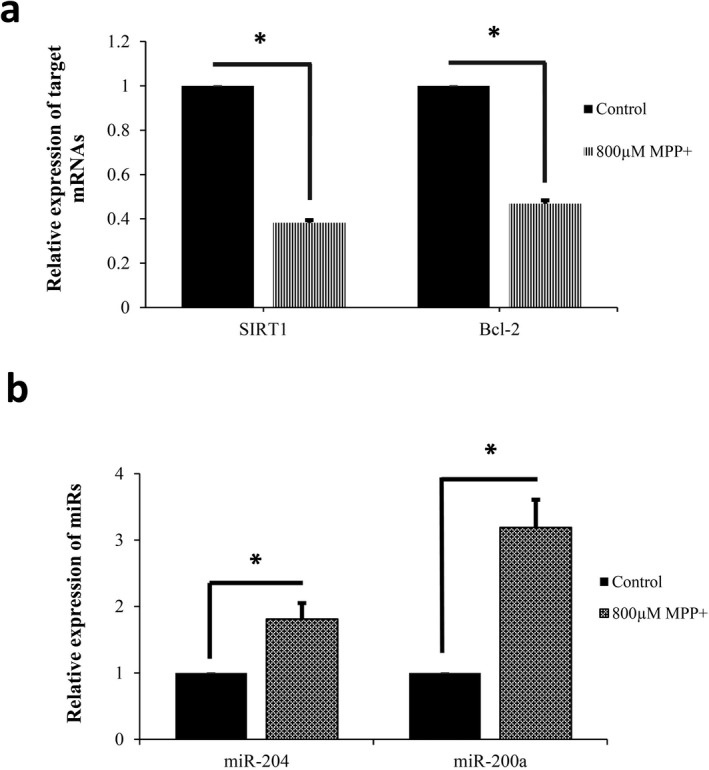
Downregulation of target genes and upregulation of miRNAs in differentiated PC12 cells exposed to MPP^+^. (a) Target genes expression were measured by real‐time RT‐PCR showing that mRNA levels of* SIRT1* and *BCL2* were found to be remarkably declined in response to MPP^+^.The relative expression of these genes was normalized by *Gapdh *as the reference gene. (b) The expression levels of miRNAs were assessed by RT‐qPCR representing miR‐200a and miR‐204 levels were remarkably higher in the cells treated with MPP^+^ compared to control group. As a reference gene, U6 snRNA was used for normalization of miRNA expression. (*p < 0.05, independent‐samples *t* test)

In order to examine changes in selected genes expression, RT‐qPCR was carried out demonstrating that *Sirt1* and *BCL2* expression was significantly affected by oxidative stress. The transcript levels of *Sirt1* was found to be considerably decreased after exposure of differentiated PC12 cells to 800 μM MPP^+^ for 24 hr, compared to that of unexposed control. Similarly, *BCL2* was significantly downregulated after MPP^+^ treatment (Figure 5a). The expression of genes was normalized to transcript level of *Gapdh* as a reference gene.

### 3.7 Upregulation of miR‐200a and miR‐204 following MPP^+ ^exposure

Changes in miRNAs levels were also quantified using real‐time PCR and expressions were normalized to U6 snRNA level. Treatment of SHSY‐5Y cells with 800 μM MPP^+^ for 24 hr led to a remarkable increase in miR‐200a level compared to untreated cells as a control group. Similar to miR‐204a, miR‐200a was remarkably upregulated after exposure to MPP^+^ (Figure 5b).

## 4 DISCUSSION

The search for understanding molecular mechanisms implicated in PD to define novel therapeutic strategies is an active area of research with a large number recently published studies investigating the use of different genes and miRNAs as potential biomarkers for the disease. Moreover, restricted availability of targeted tissues as a limitation in PD research made us to use a known cellular PD model as an alternative for these difficult‐to‐access samples (Delavar et al., 2018; Kanagaraj et al., 2014). Accordingly, we evaluated differences in genes and miRNAs expression in MPP^+^‐intoxicated differentiated PC12 cells as a PD model.

The principal mechanisms of MPP^+^ toxicity include induction of oxidative stress and mitochondrial dysfunction ultimately leading to apoptotic cell death (Farshbaf et al., 2016). Consistent with previous studies on MPP^+^‐induced cell death mechanisms, data from this study showed that MPP^+^ exposure caused a dose‐dependent reduction in cell viability, induced apoptosis, and enhanced ROS overproduction (Chen et al., 2006; Farshbaf et al., 2016; Lipman et al., 2006; Zheng et al., 2016).

There are controversial findings around *SIRT1* changes in response to MPP^+^. Here, mRNA level of* Sirt1* was found to be remarkably declined in response to MPP^+^ (Figure 5a). Consistently, the level of *Sirt1* is markedly downregulated in toxic models of PD (Dong et al., 2016; Pallas et al., 2008). In contrast, levels of *SIRT1 *increase as a protective response to neurodegenerative conditions in some models of neurodegeneration (Kim et al., 2007). According to the results of previous reports, reduced levels of *SIRT1* can be regarded as a feasible factor to pursue neural loss arising from acute stress and a reliable sensor of neurotoxic process, for example, neurodegeneration (Pallas et al., 2008).

Conflicting results were reported about *BCL2* alterations in different PD models. Here, we indicated that MPP^+^ caused significant reduction in *BCL2 *in differentiated PC12 cells (Figure 5a). Consistent with our result, numerous studies demonstrated reduction in *BCL2* in response to MPP^+^ (Chen et al., 2006; Zheng et al., 2016). In contrast, the significant enhancement of *BCL2* expression has been reported in some PD models as a compensatory mechanism (Veech et al., 2000).

Collectively, a remarkable decline in *Sirt1* and *BCL2 *levels in response to MPP^+^ demonstrates that cells lose their capability to respond rapidly and provide protection against acute oxidative stress (Pallas et al., 2008), suggesting the association of these genes with MPP^+^ toxicity mechanisms.

miR‐200a is downregulated and shows tumor‐suppressive properties in different cancer types (Tsai et al., 2011). Here, quantification of miR‐200a expression was carried out by RT‐qPCR representing miR‐200a level was remarkably higher in the cells treated with MPP^+^ compared to control group (Figure 5b). In agreement with our results, the expression level of miR‐200a, a known redoximiR, was significantly elevated by H2O2‐induced oxidative stress in hepatic, endothelial, and ovarian cancer cells (Xiao et al., 2015). Some reports revealed an enhanced expression of miR‐200a in A53T‐transgenic mice and PD patient's CSF (Mo et al., 2017). Additionally, the upregulation of miR‐200a has been confirmed in Huntington's disease (HD) model and blood samples of AD patients (Sinha, Mukhopadhyay, & Bhattacharyya, 2012; Wu et al., 2016). miR‐200a has been also identified as a plasma‐based circulating miRNA biomarkers for PD (Khoo et al., 2012). miR‐200a‐3p is upregulated in the hippocampus of APPswe/PSDE9 mice as a model of AD (Zhang, Liu, & Lu, 2017).

miR‐204 is one of the known tumor suppressors found to modulate apoptosis and be remarkably downregulated in many types of cancers (Surgucheva, Gunewardena, Rao, & Surguchov, 2013). Here, we demonstrated that similar to miR‐200a, the expression level of miR‐204 was increased in differentiated PC12 cells following MPP^+^ treatment (Figure 5b). Consistently, miR‐204 was found to be significantly upregulated in HD patients brain and AD patients CSF (Maciotta et al., 2013; Sinha et al., 2012). Moreover, miR‐204 was among upregulated miRNAs with age in mouse hippocampal neurons (Mohammed et al., 2016).

Altogether, opposite expression pattern observed between miR‐200a and miR204 with target genes can be regarded as a confirming evidence for our predicted interactions. Although MPP^+^ neurotoxicity mechanisms are not fully understood, they can include various signaling messengers, such as ROS, PI3k, and cAMP leading to considerable alterations in genes expression (Lipman et al., 2006). Thus, MPP^+^ can promote apoptosis by inducing expression of some proapoptotic miRNAs like miR‐200a and miR‐204 and subsequently affect protective genes expression here. As reported by Li, Xyu, Liu, Liu, & Wang (2015), overexpression of miR‐204 promotes apoptosis through suppressing expression of *BCL2* and *SIRT1*. According to Zhang et al. (2017), miR‐200a‐3p promotes b‐Amyloid‐induced neuronal apoptosis through downregulation of *SIRT1* in Alzheimer's disease. Salimian, Peymani, Ghaedi, & Esfahani, (2018), also showed that modulation in miR‐200a/*SIRT1*axis is strongly associated with apoptosis in MPP^+^‐induced PD model.

Here, MPP^+^‐induced upregulation of redoximiRs such as miR‐200a and miR‐204 can lead to downregulation of antioxidant genes like *SIRT1* and intensified oxidative stress. According to Xiao et al., miRNAs are crucial factors for modulating the oxidative stress response. For example, the induction of miR‐200a regulates the H_2_O_2_‐induced oxidative stress response and enhances H2O2‐induced cell death in different types of cell (Xiao et al., 2015). As mentioned by Chen et al., stress conditions such as aging and oxidative stress suppresses *SIRT1* expression or activity through miRNAs. In addition, several studies have provided evidence that *SIRT1* is the direct target of miR‐204 and miR‐200a in Human, but none of these interactions have yet been validated in Rat organism (Chen, Shentu, Wen, Johnson, & Shyy, 2013).

Taken together, it is demonstrated for the first time that miR‐204 and miR‐200a upregulates in MPP^+^‐intoxicated differentiated PC12 cells. Aberrant expression of studied miRNAs and target genes observed here is highlighting their potential significance and relevance to PD‐related mechanisms containing apoptosis and oxidative stress. Moreover, we obtained three miRNA–mRNA pairs showing opposite expression pattern in MPP^+^‐induced oxidative stress model. However, additional in vitro and in vivo experiments are also needed to verify our target prediction analysis.

## CONFLICT OF INTEREST

None of the authors has any conflict of interests to disclose and all authors support submission to this journal.
