# Supplementary figures and images for: Upregulation of miR‐200a and miR‐204 in MPP+‐treated differentiated PC12 cells as a model of Parkinson’s disease
Source: Mol Genet Genomic Med. 2019 Feb 3;7(3):e548. doi: 10.1002/mgg3.548 (PMC6418372; doi:10.1002/mgg3.548)

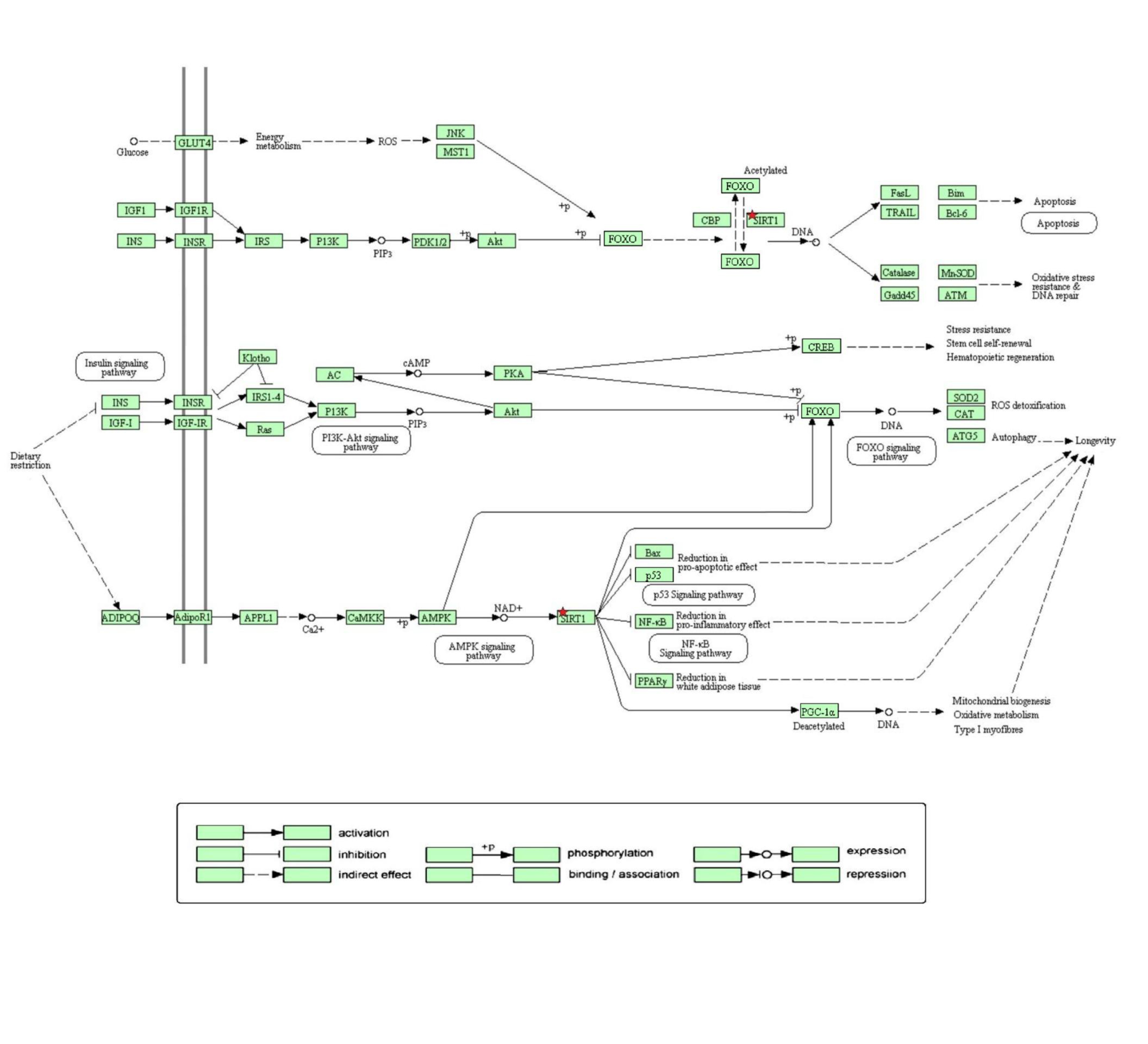

Supplement: Supplementary file 1 [file MGG3-7-na-s001.jpg]

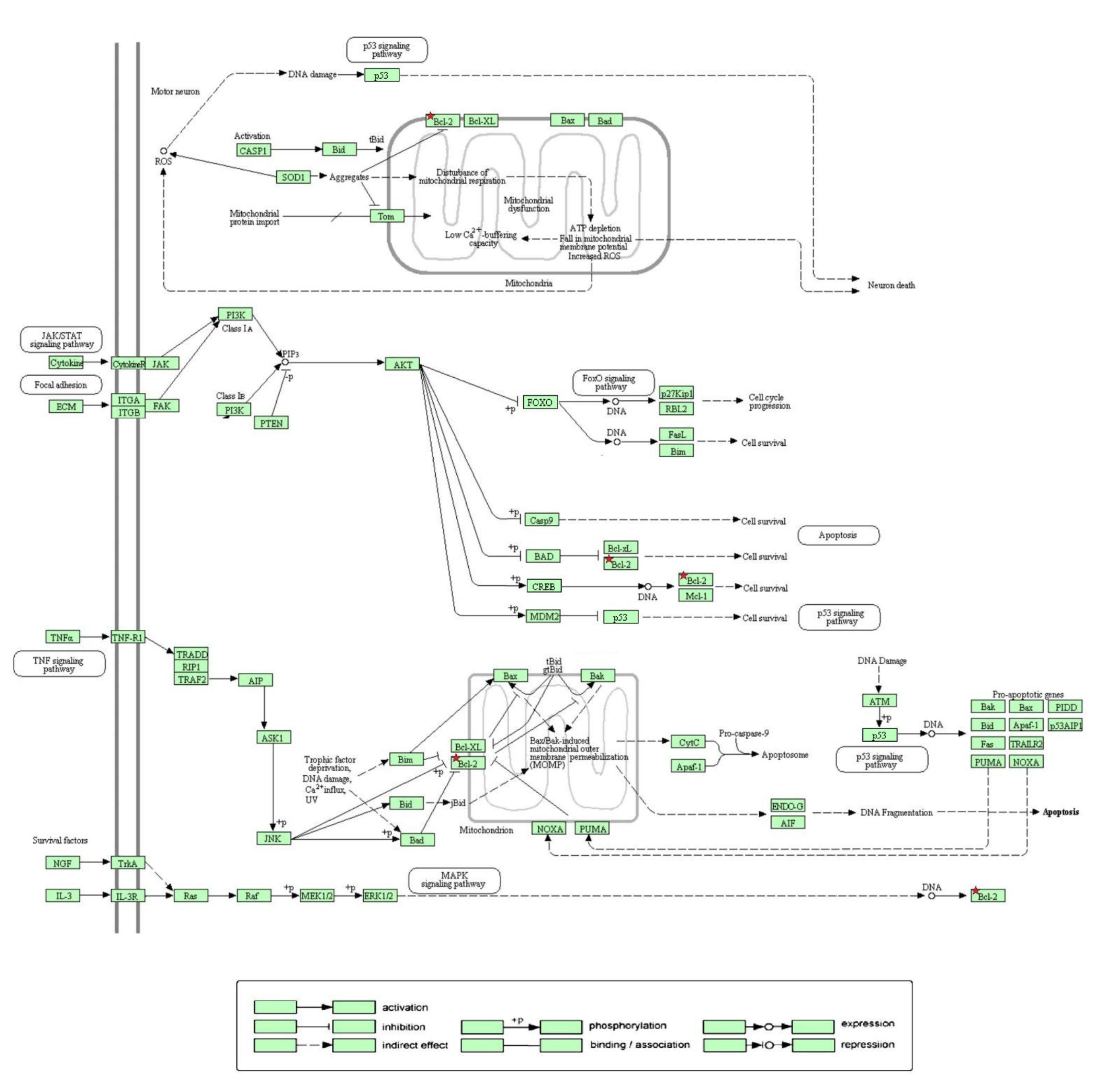

Supplement: Supplementary file 2 [file MGG3-7-na-s002.jpg]
